# Leveraging on digital technology for financial inclusion of women agripreneurs in Southern Nigeria

**DOI:** 10.12688/f1000research.122123.2

**Published:** 2023-05-16

**Authors:** Ugwuja Vivian Chinelo, Ekunwe Peter Ayodeji

**Affiliations:** 1 Agricultural Economics and Extension, University of Port Harcourt, Port Harcourt, Rivers State, 500001, Nigeria

**Keywords:** Women Agripreneurs; Digital Finance; Digital Financial Products and Services; Micro-insurance

## Abstract

Background

Women are key players in agriculture, but they are under-resourced, particularly in terms of finance. Microfinance has long been recognized as the most effective method of financially empowering these women, but using the benefits of digital technology can help scale it up and ensure its long-term viability.

Methods

The study area was Southern Nigeria. Respondents were women agripreneurs (n=479), from six states. 239 women agripreneurs who accessed digital financial products and 240 women agripreneurs who did not access financial products participated in the survey in 2019.

Results

The tests for significant difference between income of participants and non-participants in digital finance indicated a T-value of 3.214 (P< 0.001), which implies that there was a significant difference in the income of those that are accessing digital financial products (DFPs) and those that are not accessing DFPs. The tests for significant difference between savings of participants and non-participants indicated a T-value of 2.479 (p<0.05), which also implies that there was a significant difference in the women agripreneurs’ savings for participants and non-participants in DFPs. Only 2.5% of women agripreneurs are participating in micro-insurance in Southern Nigeria.

Conclusions

Women agripreneurs who are accessing digital financial products earned more income and saved more than those who are not accessing digital financial products. This implies that you are more advantaged in using digital finance in business. Micro-insurance is poorly accessed in Nigeria, and awareness of insurance products is moderately low. This study recommends that Central Bank of Nigeria should engage in more outreach programmes to enable all women in Nigeria access digital financial products because of its convenience and contributions to success in business. Insurance companies should capitalize on business models that incorporate mobile technologies in order to increase insurance penetration in rural areas.

## Introduction

Information and communication technologies (ICTs) are a broad category of technological tools and resources used to create, disseminate, preserve, add value to, and manage data. Telecommunications, television and radio broadcasting, computer hardware, software services, and electronic media are all part of the ICT sector. ^1^ Microfinance relies heavily on information. Microfinance institutions (MFIs) collect and keep a massive amount of vital business data, ranging from basic client information to in-depth analysis of portfolio statistics. ICTs now have a greater impact on the structure and operations of businesses than any previous technology.

Financial inclusion is a critical factor in the economic development of any nation, and Nigeria is no exception. In recent years, there has been a growing recognition of the importance of digital finance as a means of promoting financial inclusion, particularly among farmers in rural areas. In Southern Nigeria, many farmers face significant challenges accessing financial services, which limits their ability to invest in their farms, increase productivity, and improve their livelihoods.

According to a report by Ref. 2, only 39.7% of Nigeria’s adult population had access to formal financial services, with a significant gap between urban and rural areas. The report also highlighted the challenges faced by farmers in rural areas in accessing financial services, including limited physical access to banks and other financial institutions, high transaction costs, and a lack of financial literacy and awareness.

The theoretical foundation of digital technology for financial inclusion is based on the concept of “digital financial services” (DFS), which refers to the use of digital technology to provide financial services. DFS can be delivered through various channels, such as mobile phones, the internet, and point-of-sale devices. The key benefits of DFS are that they are cost-effective, scalable, and can reach remote and underserved areas.

One of the most influential theories in this field is the “Technology Acceptance Model” (TAM), which was first proposed by Ref. 3. TAM posits that the adoption of technology is determined by two factors: perceived usefulness and perceived ease of use. Several studies have applied TAM to the adoption of DFS, including a study by Ref. 4 in Nigeria and a study by Ref. 5 in Zimbabwe.

Empirical research on digital technology for financial inclusion has focused on two main areas: the impact of DFS on financial inclusion outcomes and the factors that influence the adoption of DFS by consumers. A recent meta-analysis by Ref. 6 synthesized the findings of 126 impact evaluations of DFS interventions in developing countries. The analysis found that DFS interventions have a positive impact on financial inclusion outcomes, such as access to formal financial services, savings behavior, and income.

Factors that influence the adoption of DFS by consumers include demographic characteristics, such as age, gender, and education level, as well as trust in financial institutions and perceptions of the usefulness and ease of use of DFS. Several studies have also examined the role of regulatory frameworks and infrastructure in promoting the adoption of DFS, such as a study by Ref. 7 in Kenya.

Women are key players in agriculture, but they are under-resourced, particularly in terms of finance. Microfinance has long been recognized as the most effective method of financially empowering these women, but using the benefits of digital technology can help scale it up and ensure its long-term viability.

This new wave of digital finance presents tremendous opportunity for the financial sector and customers alike, promoting individual well-being and nationwide financial inclusion. Using the same dataset as this research, the authors have previously reported on a variety of digital financial products and services. ^8^ Digital financial products and services made available through digital finance are ATM/debit cards, SMS alert services, USSD banking codes, point of sale (POS), balance inquiry, online fund transfer, email alerts, online bill payments, e-statements, online purchases, remittances, online loans, online deposit accounts, online savings accounts and micro-insurance. According to the authors’ previous research, ^8^ micro-insurance notwithstanding its benefit is the least accessed digital financial product. Micro-insurance protects the agripreneurs against losses caused by crop failure. It acts like a tool that allows farmers to manage their yield and price risks. Farmers are able to repay their loans even during the time of crop failure with the support of the right insurance partner.

Despite improvement in financial technology, women remain underrepresented among the banked and formally served, and women remain significantly excluded compared to men, ^9^ Enhancing Financial Innovation and Access (EFINA) ^9^ also indicated that 35 percent of Nigerian rural women have no bank account compared with 60 percent of urban women. The majority of these rural women are agripreneurs whose main occupation is mostly farming.

Some studies have identified financial availability and accessibility as one of the primary impediments and restrictions to economic progress. ^10^ Women as a group who are usually available and willing to embark on entrepreneurial ventures, are hindered sometimes due to the following factors; absence of start-up capital, lack of awareness of existing credit schemes; high interest rates; long and rigorous processes for loan applications; and lack of collateral security for loans. ^10^

When we talk about agripreneurship, we refer to the totality of activities which include making profit through commercializing different types of agricultural produce. ^11^ This will range from earning income from farming activities like crop cultivation to fish farming and animal husbandry. ^12^ An agripreneur is a risk-taker, an imaginative and creative genius who has the ability to design and introduce new products capable of drawing customer’s attention. Agripreneurs are productive and can spot unique business opportunities as they look for better methods to organise their farms, try new crops and cultivars, breed better animals, and use alternative technology to boost productivity, diversify production, minimise risk, and improve profit.

According to a study embarked upon by Ref. 13, women entrepreneurs, given their position and educational level, could have fantastic business ideas but would lack the requisite capital to executive those ideas. In their view, if they were provided with the right assistance financially and the right guidance, these women could transform to labour employers in no time. The scholars advanced their study by positing that “before the coming of the colonial administrations, African women had led the way, or at least played important roles, in the social and economic development of their different traditional communities”. Nevertheless, the injection of a Victorian culture or idea where women were sidelined in the public organization of things brought about women’s role marginalization. The result in Nigeria became that women could not have a direct access to credit despite the amount involved. Many of them would have their husband, father or brother guarantee them before they got the loan ^14^ also found similar evidence in Latin American communities, that aspiring women entrepreneurs were less likely to access formal credit than men were.

Women typically do not have access to assets or family property, which contributes to their poverty. It is necessary to conduct a study that evaluates their bad state in order to formulate appropriate policy. Hence the need for this study.

### Objectives of the study

The specific objectives of this study were to:

i. identify conditions for accessing digital financial products and services (DFS) among women agriprenuers in Southern Nigeria;
ii. compare assets, income and savings of women agriprenuers with and without digital financial products and services in Southern Nigeria;
iii. examine the level of awareness, perception and participation of women agripreneurs in micro-insurance schemes in Southern Nigeria.

### Hypothesis of the study

**Ho**_**1**_: There is no significant difference in assets, income and savings of women agripreneurs with and without digital financial products and services (DFS) in Southern Nigeria.

## Methods

### Ethics and consent

The study was reviewed and approved by the University of Port Harcourt Research Ethics Committee on 2/2/2018 to make sure the research meets high ethical and scientific standards. Verbal consent was obtained from the women agripreneurs; a consent form was attached to the questionnaire which every participant verbally agrees to before participating in the research, this was approved by ethics committee. The reason for verbal consent is because this is the form of consent we obtain when some of the respondents are not literate. The purposes and importance of this study were explained to all women agripreneurs. The responses of each respondent were kept confidential by coding. The data were collected and analyzed anonymously.

### Area of study

The area of the study was Southern Nigeria. It has a population of 64,978,376 people and covers a total land area of 193,347 km2 (NPC, 2006). Nigeria is divided into six geopolitical zones, three of which make up Southern Nigeria. It is made up of 17 states out of Nigeria's 36. It is covered by a diverse range of vegetation belts, from Nigeria's largest rain forests to mangrove swamps, savannahs, mountains, and waterfalls, all of which are teeming with rare animals, endangered species, and unusual plant families, making it one of the world's richest biodiversity hotspots, attracting both scientists and tourists. The Niger Delta is a Southern Nigerian. This is where the lion's share of the country's oil is discovered.

### Sampling procedure and sampling size

A multistage sampling procedure was used to choose the respondents for this study. Southern Nigeria is divided into three geopolitical zones: southeast (five states), southwest (six states), and south-south (six states). In each geopolitical zone, two states were chosen at random using simple randomization method, making a total of six states for the study. Abia, Enugu, Bayelsa, Rivers, Ekiti, and Ondo were the states selected. Two Local Government Areas (LGAs) were purposively picked in each selected state, giving a total of 12 LGAs. The LGAs in Abia were Umuahia North and Umuahia South LGAs, in Bayelsa, Yenagoa and Sagbama LGAs, in Enugu, Orji River and Nkanu West LGAs, in Rivers, Ikwerre and Khana LGAs, in Ekiti, Ikere and Ado-Ekiti LGAs, and in Ondo, Akure North and Ifedore LGAs. The study selected two farming communities purposively from each LGA, totaling 24 farming communities. Purposive selection was made based on the presence of financial institutions in the farming communities. Enumerators, who are professional data collectors, assisted in gathering the participants, using key informants in the selected communities. Respondents were approached in their farms, shops, homes and their meeting venues. In each selected community, there was a purposive selection of ten women agripreneurs who use digital financial products and ten women agripreneurs who don't use digital financial goods. This gave a total of 240 of them who have access to digital financial products, and another 240 who do not have access to financial products. For the entire survey, there were 480 women agripreneurs.

### Data collection

Primary data were collected starting from 26/10/2018, using structured questionnaires where literate participants filled in the questionnaire themselves, and for illiterate participants an oral interview was conducted with the help of enumerators filling the questionnaire on their behalf. The research instrument was validated by a panel of experts in Agricultural Economics and Cybersecurity to make sure it possessed both face and content validity. The researchers ensured that all the corrections pointed out were incorporated before making the final draft. The study used two sets of questionnaires: one for women agripreneurs who use digital financial products, and another for women agripreneurs who do not. The questionnaires have open ended and Yes/No questions.

### Data analysis

The data were analyzed using two distinct approaches: descriptive statistics and inferential statistics such Z-Test. Objectives i and iii were achieved using descriptive statistics such as mean, frequencies and percentages. The software that was used for analysis is SPSS version 25 (2017).

### Model specification for Z-test model

Objective ii was achieved using Z-test. The analysis was done separately for assets, income and savings comparing these variables for women agripreneurs that are using digital financial products and services and those that are not using digital financial products and services. The Z –statistic is mathematically specified as;

$$Z=\frac{\hat{X}-\hat{Y}}{\sqrt{\frac{{S^{2}}_{x}}{n_{x}}+\frac{{S^{2}}_{y}\;}{n_{y}}\;}}\;$$ (1)

*Z* = the value by which the statistical significance of the mean difference would be judged

$\hat{X}$ = Mean amount of assets/income/saving women agripreneurs that are accessing digital financial products and services (DFS)

$\hat{Y}$ = Mean amount of assets/income/savings of women agripreneurs that are not accessing DFS

*S* ^2^_*x*_ = Variance of mean amount of assets/income/savings of women agripreneurs that are accessing DFS

*S* ^2^_*y*_ = Variance of mean amount of assets/income/savings of women agripreneurs that are not accessing DFS

*n*_*x*_ = Sample size of women agripreneurs that are accessing DFS

*n*_*y*_ = Sample size of women agripreneurs that are not accessing DFS

## Results and discussion

### Conditions for accessing digital financial products and services among women agripreneurs in Southern Nigeria

**Figure 1.**
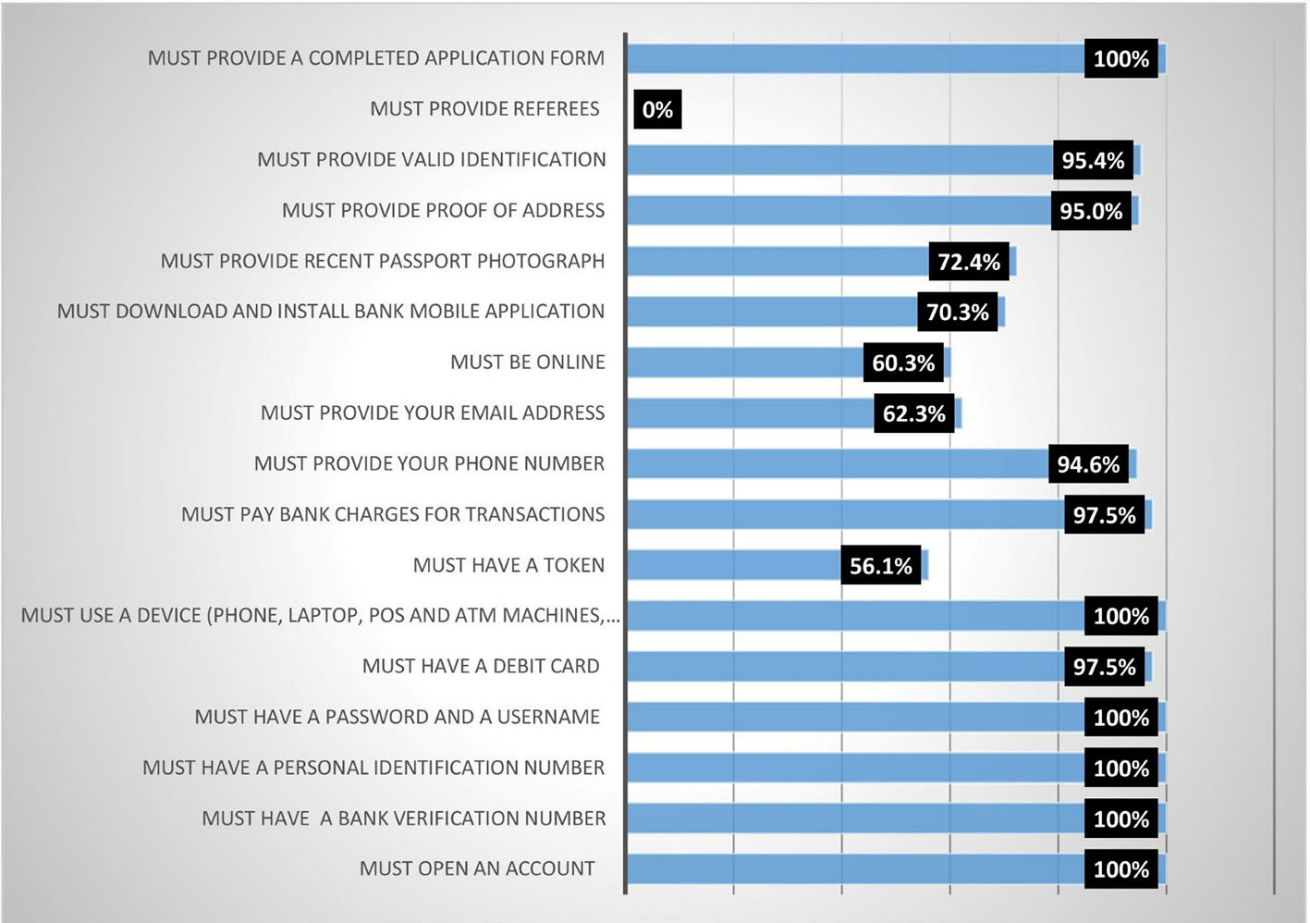
Conditions for accessing digital financial products.

Results from Figure 1 show that all (100%) the female heads agreed that they must meet the following conditions before they could access digital financial products (DFPs) (1) Must provide a completed application form; (2) Must use a device (phone, laptop, point of sale (POS) and ATM machines, etc.); (3) Must have a password and a username; (4) Must have a personal identification number; (5) Must have a bank verification number; (6) Must open an account. Finding 2 conforms with expectations because digital financial products are accessed through electronic devices. The majority (95.4%) of the respondents indicated that you must provide a valid identification card, while 94.6% indicated that you must provide your phone number. About 72.4% of the respondents agreed that you must provide a recent passport photograph, while 70.3% agreed that you must download and install a mobile bank application. This finding corroborates with the report of Ref. 15 who stated that Palestinian bank customers accessed digital financial products and services through ATMs and mobile banking applications.

Additionally, analysis on the results shows that respondents agreed moderately with the following conditions: 1) You must be online (60.3%); 2) You must provide your email address (62.3%); 3) You must have a token (56.1%). None (0%) indicated that you must provide a referee. Participants agreed that they were subjected to the indicated conditions before accessing digital financial products and services.

### Income, savings and assets differentials among women agripreneurs who accessed digital financial products and those that did not access them

In Table 1, the different means of income, savings and assets of women agripreneurs that are accessing DFPs and those that are not accessing are presented. The tests for significant difference between income of participants and non-participants indicated a T-value of 3.214 (p<0.001), which implies that there was a significant difference in the annual income of those that are accessing DFPs and those that are not accessing DFPs. The tests for significant difference between annual savings of participants and non-participants indicated a T-value of 2.479 (p<0.05), which also implies that there was a significant difference in the women agripreneurs’ savings for participants and non-participants in DFPs. The tests for significant difference for assets between participants and non-participant in DFPs was not significant. The mean annual income was NGN372,938.22 and NGN288,720.06 for participants and non-participants respectively, and mean annual savings were NGN132,534.23 and NGN86,304.17 for participants and non-participants respectively. This implies that women agripreneurs who accessed DFPs had more successful businesses than those who did not access DFPs.

**Table 1.** Results of the Z-test comparing income, savings and assets of participants and non-participants in digital financial products (DFPs).

Statistic measures	Annual income		Annual savings		Assets	
	Participants	Non-participants	Participants	Non-participants	Participants	Non-participants
Sample size	239	240	239	240	239	240
Mean	NGN372,938.22	NGN288,720.06	NGN132,534.2	NGN86,304.17	3.91	3.51
T-ratio	3.214		2.479		1.543	
P-Value	.001		.014		.124	

Source: Field survey, 2019.

### Awareness of micro-insurance schemes by women agripreneurs in Southern Nigeria

**Figure 2.**
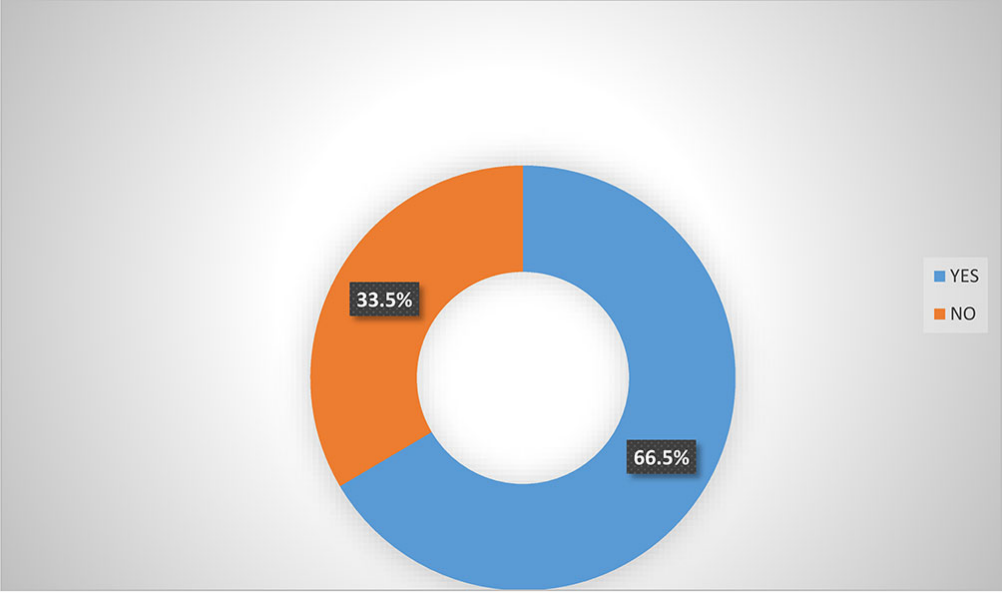
Awareness of micro-insurance schemes by women agripreneurs that are accessing digital financial products.

**Figure 3.**
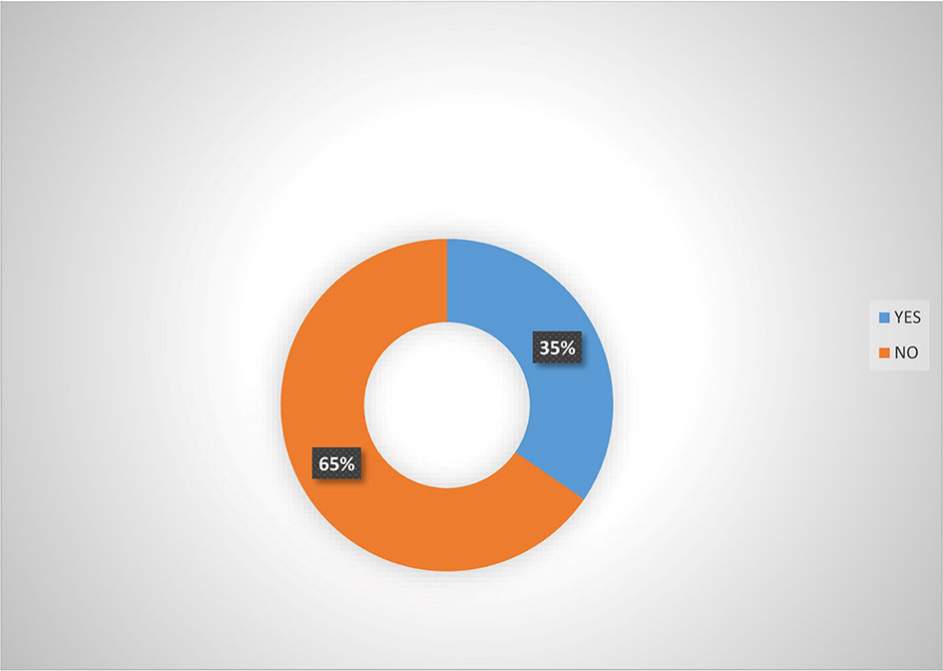
Awareness of micro-insurance  by women agripreneurs that are not accessing digital financial products.

**Figure 4.**
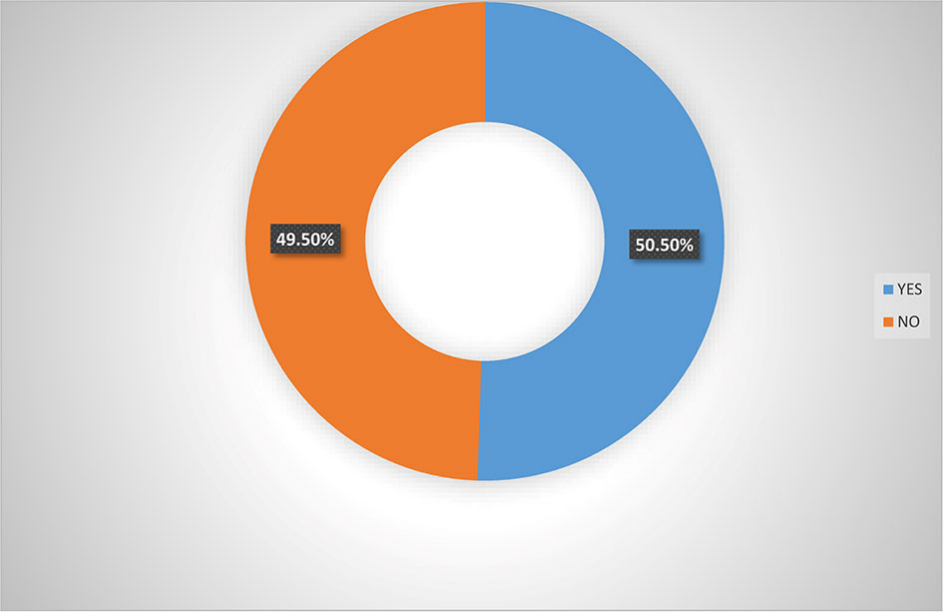
Pooled responses on awareness of micro-insurance by women agripreneurs.

Analysis from Figure 2 shows that majority (66.5%) of women agripreneurs that are accessing digital financial products agreed that they are aware of micro-insurance schemes in Southern Nigeria. Analysis from Figure 3 also indicated that majority (65.0%) of women agripreneurs who are not accessing digital financial products indicated that they are not aware of micro-insurance schemes, implying that women agripreneurs who access digital financial products know more about micro-insurance than those who are not accessing digital financial products. Pooled response from Figure 4 indicated that 50.5% of the women agripreneurs in Southern Nigeria are aware of micro-insurance schemes in Nigeria, implying that on average half of the respondents are aware of micro-insurance schemes.

### Participation in micro-insurance schemes by women agripreneurs in Southern Nigeria

**Figure 5.**
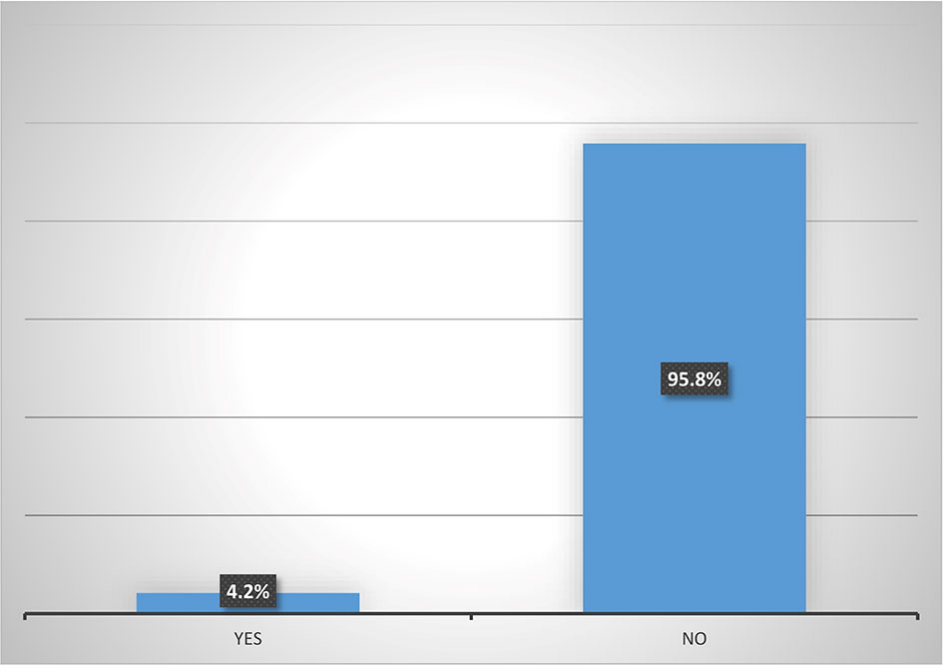
Participation in micro-insurance by women agripreneurs that are accessing digital financial products.

**Figure 6.**
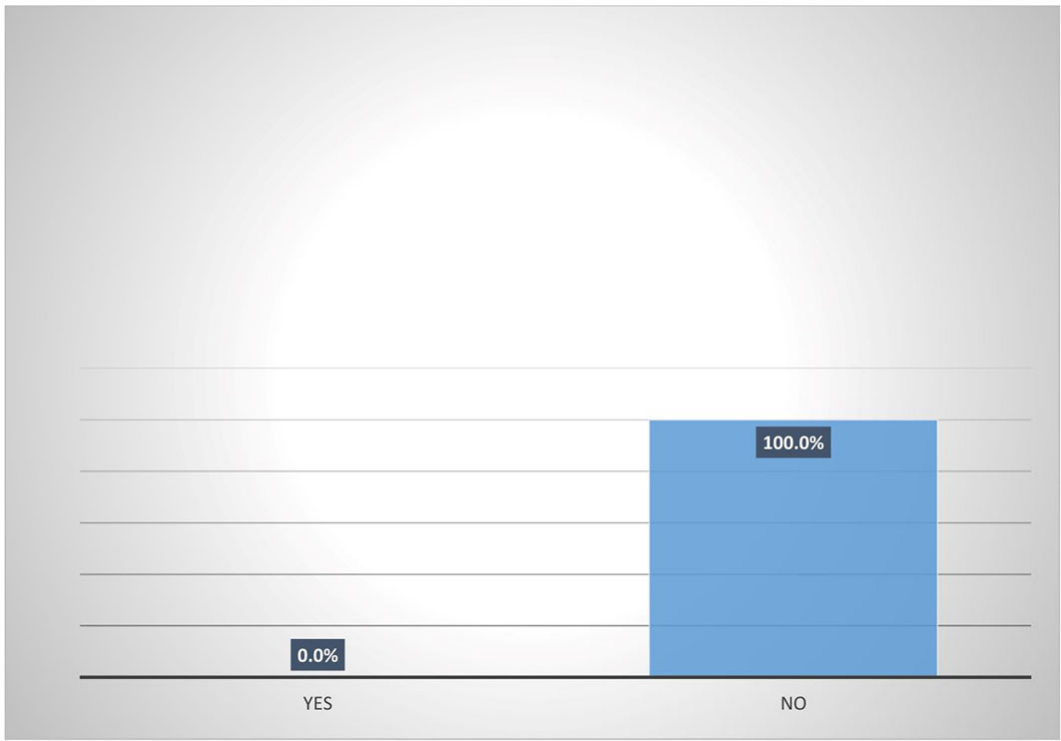
Participation in micro-insurance by women agripreneurs that are not accessing digital financial products.

**Figure 7.**
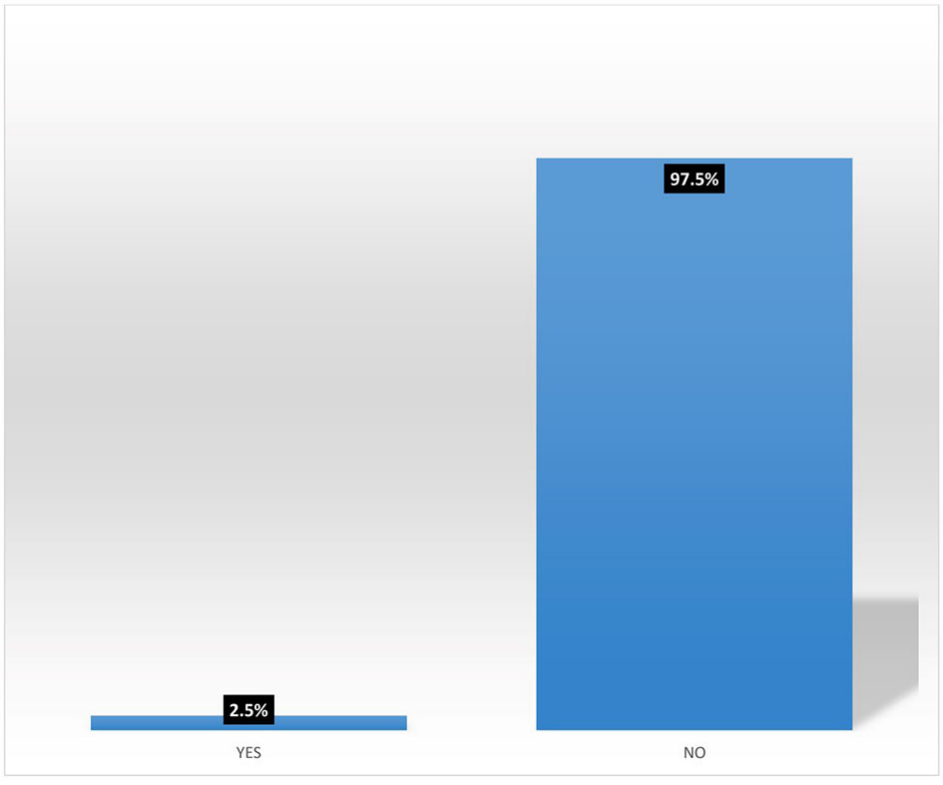
Pooled responses on participation in micro-insurance by women agripreneurs.

Results from Figure 5 shows that majority (95.8%) of female heads who are accessing digital financial products are not participating in micro-insurance schemes. Also, Figure 6 shows that none (0%) of the female heads who are not accessing digital financial products participates in micro-insurance schemes. Figure 7 shows pooled responses from women agripreneurs who are accessing and not accessing digital financial products on participation in micro-insurance schemes. Only 2.5% of the respondents are participating in micro-insurance. The implication of this is that many Nigerians do not access micro-insurance products. This corroborates the findings of Ref. 16 which states that out of 96.4 million adults, only 0.3 million use micro-insurance products. The findings of Ref. 8 also reported poor participation of women in accessing insurance products.

### Perceptions of women agripreneurs on insurance products

**Figure 8.**
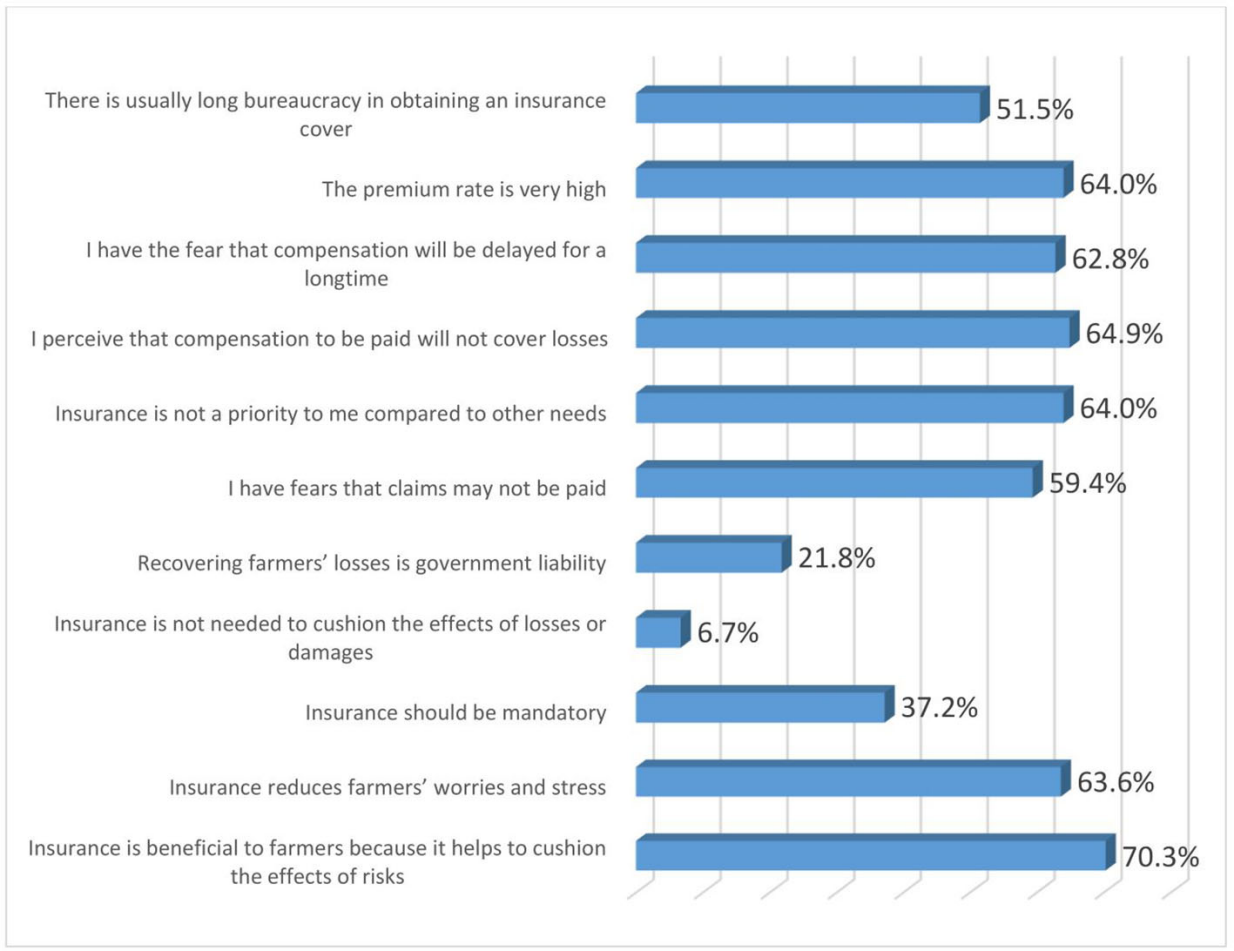
Perceptions of women agripreneurs who are accessing digital financial products on insurance.

Figure 8 shows the perceptions of women agripreneurs who are accessing digital financial products on insurance. Most of them agreed with the following statements: (1) Insurance is beneficial to farmers because it helps to cushion the effects of risks (70.3%); (2) I perceive that compensation to be paid will not cover losses (64.9%); (3) The premium rate is very high (64%); (4) Insurance is not a priority to me compared to other needs (64%); (5) Insurance reduces farmers’ worries and stress (63.6%);(6) I have the fear that compensation will be delayed for a long time (62.9%); (7) I have fears that claims may not be paid (59.4%) and (8) There is usually long bureaucracy in obtaining an insurance cover (51.5%).

A minority of the respondents agreed to the following statements: (1) Insurance is not needed to cushion the effects of losses or damages (6.7%); (2) Recovering farmers’ losses is government liability (21.8%); (3) Insurance should be mandatory (37.2%).

**Figure 9.**
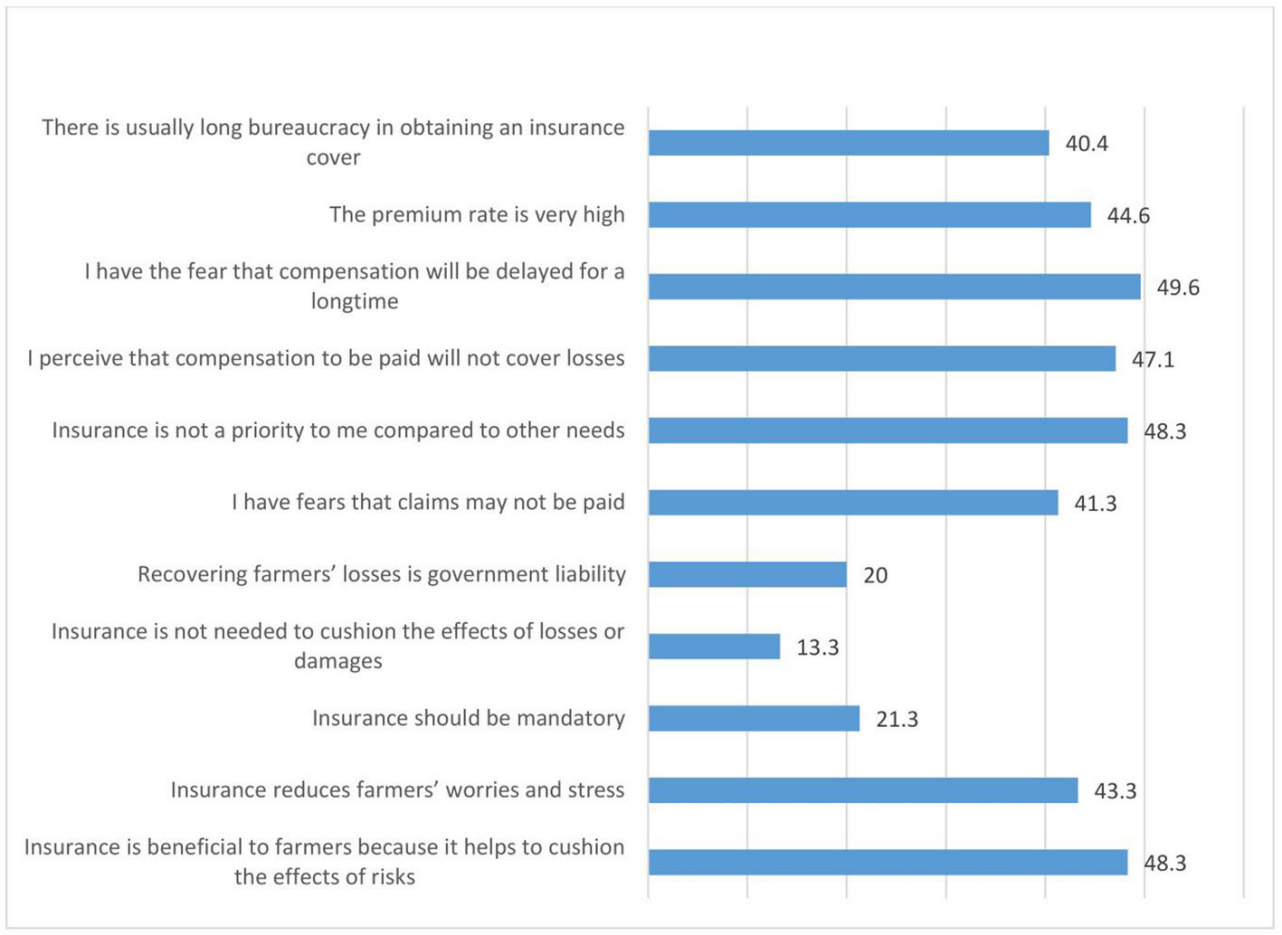
Perceptions of women agripreneurs who are not accessing digital financial products on insurance.

Results from Figure 9 shows the perception of women agripreneurs who are not accessing digital financial products on insurance. They agreed moderately to the following statements: (1) I have the fear that compensation will be delayed for a long time (49.6%); (2) Insurance is not a priority to me compared to other needs (48.3%); (3) Insurance is beneficial to farmers because it helps to cushion the effects of risks (48.3%) (4) I perceive that compensation to be paid will not cover losses (47.1%); (5) The premium rate is very high (44.6%); (6) Insurance reduces farmers’ worries and stress (43.3%); (7) I have fears that claims may not be paid (41.3%); (8) There is usually long bureaucracy in obtaining an insurance cover (40.4%).

A minority of the respondents agreed with the following statements: (1) Insurance is not needed to cushion the effects of losses or damages (13.3%); (2) Insurance should be mandatory (21.3%); (3) Recovering farmers’ losses is government liability (20.0%).

From the findings of this research, the perceptions of these women on micro-insurance could be a major reason why they don’t participate in insurance schemes.

## Conclusion

Digital finance through the use of financial technology may be very convenient but has many conditions required to access it. Findings from this study shows that users must use a device (phone, laptop, POS and ATM machines, etc.), must have a password and a username, must have a personal identification number, must have a bank verification number and you must open an account. Women agripreneurs who are accessing digital financial products earned more income and saved more than those who are not accessing digital financial products. This implies that you are more advantaged in using digital finance in business. Micro-insurance is poorly accessed in Nigeria, and awareness of insurance products is moderately low. Most of the women agripreneurs perceive that insurance is beneficial to farmers because it helps to cushion the effects of risk, and also perceive that compensation to be paid will not cover losses and that the premium rate is very high. This study recommends that Central Bank of Nigeria should engage in more outreach programmes to enable all women in Nigeria access digital financial products because of its convenience and contributions to success in business. Insurance companies should capitalize on business models that incorporate mobile technologies in order to increase insurance penetration in rural areas. This is the first research that has studied digital finance and perception on insurance of women agripreneurs in Southern Nigeria. A limitation is that it was not able to study men agripreneurs, and therefore it is recommended that future research should be carried out on the digital finance and perception on insurance of men agripreneurs. The government should provide subsidies and incentives to farmers who adopt digital financial services. This can be done by providing reduced transaction fees or cash-back rewards for farmers who use digital financial services to save or invest in their farm. Government can encourage partnerships between financial institutions and agribusinesses to promote the use of digital financial services among farmers. This can be done by providing incentives for financial institutions to partner with agribusinesses and by creating platforms that facilitate these partnerships.

## Data availability

Underlying data figshare: Leveraging on Digital Technology for Financial Inclusion of Women Agripreneurs in Southern Nigeria. https://doi.org/10.6084/m9.figshare.19657671.v1 ^17^

This project contains the following files

- Non participants.sav (raw data file)
- Participants.sav (raw data file)

### Extended data

figshare: Leveraging on Digital Technology for Financial Inclusion of Women Agripreneurs in Southern Nigeria. https://doi.org/10.6084/m9.figshare.19657671.v1 ^17^

This project contains the following files

- Questionnaire for participants.sav
- Questionnaire for non-participants.sav
- Data key for participants
- Data key for non-participants

Data are available under the terms of the Creative Commons Attribution 4.0 International license (CC-BY 4.0).
