# Supplementary material for: Leveraging on digital technology for financial inclusion of women agripreneurs in Southern Nigeria
Source: F1000Res. 2023 Dec 11;11:704. Originally published 2022 Jun 27. [Version 3] doi: 10.12688/f1000research.122123.3 (PMC10918311; doi:10.12688/f1000research.122123.3)
Supplement: Peer review history

# Reviewer report: Reviewer response for version 3

**DOI:** 10.5256/f1000research.159847.r229103

**Published:** 2024-06-14

**Authors:** Lee-Ying Tay

**Affiliations:** 1 Faculty of Business, Multimedia University, Melaka, Malaysia

The author has included all my suggestions.

Is the work clearly and accurately presented and does it cite the current literature?

Partly

If applicable, is the statistical analysis and its interpretation appropriate?

Yes

Are all the source data underlying the results available to ensure full reproducibility?

No source data required

Is the study design appropriate and is the work technically sound?

Partly

Are the conclusions drawn adequately supported by the results?

Partly

Are sufficient details of methods and analysis provided to allow replication by others?

Partly

Reviewer Expertise:

Area of research: Digital finance, Stock price, Digital financial products and services

I confirm that I have read this submission and believe that I have an appropriate level of expertise to confirm that it is of an acceptable scientific standard.

# Reviewer report: Reviewer response for version 3

**DOI:** 10.5256/f1000research.159847.r238530

**Published:** 2024-04-03

**Authors:** Rachel Hay, William Harrington, Carrie-Ann Wilson

**Affiliations:** 1 James Cook University, Townsville City, Queensland, Australia; 2 College of Business Law and Governance, James Cook University, Townsville City, Queensland, Australia; 3 College of Business, Law and Governance, James Cook University, Townsville City, Queensland, Australia

* I appreciate the opportunity to review this paper alongside my PhD students, William Harrington and Carrie-Anne Wilson.

 * Overall the topic of discussion is important and interesting

 There needs to be clearer delineation of who the data is reporting on 1) all women participants, 2) a subset of women agripreneurs - this is not clear throughout the analysis e.g., if participants who did not identify as women agripreneurs were removed, this needs to be stated in the methodology, likewise if they were kept in then see 1) and 2) above.

 * Overall, the paper requires deeper compare and contrast in the literature review to situate the research for the reader.

 * The implications to policy could be clearer

 * The statistical analysis, is appropriate, but the authors need to clearly identify limitations within the methodology.

**Abstract**

**Results Section**

 * the results of the statistical analysis implies/demonstrate what the data tells us. **It implies or demonstrates the correct language?**

* the results discuss objectives 1 and 2, but for objective 3 only participation is included, need to include results from perception and awarness

 * why is it important to know only 2.5% of women agripreneurs are participating in micro-insurance in Southern Nigeria. **This sentence is hanging, extend to identify the contribution to the objective e.g., can we attribute that to awareness, perception?  possible re-write:** While the data clearly demonstrates that only 2.5% of women agripreneurs are participating in micro-insurance in Southern Nigeria, we are unsure if this results from a lack of awareness or poor perception of the benefits of micro-insurance. 

**Body of work**

 * overall the referencing style is inconsistent, some are superscript and others are identified by Ref. # - **change to be more consistent,** consider using APA referencing. There are insufficient references.  Nearly half of the references included are grey literature reducing the credibility of the work. Use the prompts below to increase academic literature.

**Introduction**

 * Para 1 hanging sentence: "Microfinance relies heavily on information." **remove**

 * Para 1: MFI sentence **needs a reference**

 * Para 2: Financial inclusion is a critical factor in the economic development of any nation **(REF)**

 * Para 2: In recent years, there has been a growing recognition of the importance of digital finance as a means of promoting financial inclusion, particularly among farmers in rural areas **(REF)**

 * Para 4: digital financial services **(REF)** https://pubdocs.worldbank.org/en/230281588169110691/Digital-Financial-Services.pdf

 * Para 5: Several studies have applied TAM to the adoption of DFS, including a study by Ref. 4 in Nigeria and a study by Ref. 5 in Zimbabwe- what did these papers tell us - extend e.g., Sadiq et al., (2019) found that... this was supported/contrasted by Chibba (2018) who identified... then write about how this supports/directs your study.  This important critical analysis provides the reader with context around why this topic is of interest.

 * Para 6: *"Empirical research on digital technology for financial inclusion..."* is critically analysed much better

 * Para 7: Factors that influence the adoption of DFS by consumers include demographic characteristics, such as age, gender, and education level, as well as trust in financial institutions and perceptions of the usefulness and ease of use of DFS (REFERENCES). ........ such as a study by Ref. 7 in Kenya, which found.... extend this to provide context.

 * Para 8: Microfinance has long been recognized as the most effective method of financially empowering these women (REF). Digital technology can help scale....

 * Para 9: Note self-citing from grey literature - reduces credibility

 * Para 9: self reference/circular reference - citing the same data from two different resources of the same author and database does not increase the credibility of the finding - eg., According to the authors’ **previous research (From the same dataset)**, ^8^ micro-insurance notwithstanding its benefit is the least accessed digital financial product.

 * Para 10: agripreneurs - needs defining - Move paragarph "When we talk about agripreneurship, we refer to the totality of activities which include..." to after paragraph "women are key players". Start the sentence with In this study we refer to women agripreneurs, which refers to the totality of activities.....

 * Para 11: Despite improvement in financial technology, women remain underrepresented - **all women or Nigerian/African women - define**

 * para 11: sentence structure "Despite improvement in financial technology, women remain underrepresented among the banked and formally served, and women remain significantly excluded compared to men **(FULL STOP)**, ^9^ **THE **Enhancing Financial Innovation and Access (EFINA) **SURVEY (2021)** ^9^ also indicated that 35 percent of Nigerian rural women have no bank account compared with 60 percent of urban women **(pg. XX)**."  The majority **(XX%)** of these rural women are agripreneurs whose main occupation is mostly farming.

 Para 12: **Reference 10 is an unpublished masters thesis... can you find a published academic reference that can support this claim rather than relying on an unpublished masters thesis?**

 Para 13: moved to after Para 8

 Para 14: In ***their view,*** if they were provided with the right assistance financially and the right guidance, these women could transform to labour employers in no time. **whose view the author, the women entrepreneurs?**

 Para 14: The scholars advanced their study by positing that “before the coming of the colonial administrations, African women had led the way, or at least played important roles, in the social and economic development of their different traditional communities” **(pg: XX)**. **so what, why is this important?**

 Para 14: i **ntroducing a new concept - expand - **Nevertheless, the injection of a **Victorian culture or idea** where women were sidelined in the public organization of things brought about women’s role marginalization **(REF).**

 Para 14: **sentence structure - needs improving - **In ***Nigeria women cannot have a direct access to credit despite the amount involved (REF). For many, their husband, father or brother would need to guarantee them before they were approved for the loan. ^14^*** **Sabarwal et al., (2009)** also found similar evidence in Latin American communities, that aspiring women entrepreneurs were less likely to access formal credit than men were.

 Para 15: **re -write e.g.,** **In Nigeria,** women typically do not have access to assets or family property, which contributes to their poverty **(REF)**. It is necessary to conduct a study that evaluates **women's access to digital technology for financial inclusion** to formulate appropriate policy. Hence the need for this study.

**Objectives and Hypothesis are clear and concise**

**Area of Study**

 * ...It is made up of 17 states out of Nigeria's 36 **- what is Southern Nigeria, then state clearly? Southern Nigeria **is made up of 17 states out of Nigeria's 36 **and is** covered by a diverse range of vegetation belts, from Nigeria's largest rain forests... (REF).

 * **what relevance do these sentences have on the study, if none, then remove, if something then expand to identify relevance.**  "The Niger Delta is a Southern Nigerian. This is where the lion's share of the country's oil is discovered"

**Sampling Procedure and sampling size**

 * ... using simple randomization method **(REF method).**

 * ... using key informants **(REF) why were key informants used?**

* This gave a total of 240 **agripreneural women** who have access...

 * **Identify the number of participants who completed the survey who did not have access to financial products: **This gave a total of 240 **agripreneural women** who have access to digital financial products, and another 240 who do not have access to financial products, **a total of 480 potential participants.** Out of the 240 copies of questionnaire administered to respondents who have access to financial products, **239 were** successfully completed and used for analysis. **XXX number of respondent completed the survey for those who do not have access to financial products.** For the entire survey, there were 379 women agripreneurs.

**Data collection**

 * Primary data were collected starting from 26/10/2018 **and completed on XX/XX/XXXX**

 * Why were two different questionnaires used? **Extend to explain why the survey could not be completed using one survey instrument.**

**Model specification**

 * It is noted that z-tests performed in SPSS can report the wrong standard error - **if completed in spss was a manual calculation used to ensure that the analysis was correct?**

 * Cohen's H (used to measure the distance between two proportions) is not mentioned, **was Cohen's H used to test if the significance was small, medium or large?  If not, then this should be included in the results.**

**Results and Discussion**

**Overall - only a proportion were women agrientrepreneurs, the findings discussed here apply to all women participants. The authors need to discriminate with supporting data when reflecting on women agrientreprenures throughout the results and discussion.**

*Conditions for accessing digital financial products and services among women agripreneurs in Southern Nigeria*

 * Figure 1 would be better displayed as a table

* Results from Figure 1 show that all (100%) **women agripreneurs OR female respondents** agreed that **...**

* the questions about risk assume that the participant engages with online/electronic banking - a question to confirm relevance to the two groups access or not to financial products **, **would have confirmed their suitability for the study and enabled deeper analysis of the findings e.g., Do you engage in electronic banking, Do you have a valid identification card/papers, Have you downloaded and installed online banking app etc.  This data would have allowed the researchers to compare and contrast if the conditions were able to be met or that they were not met because of these limitations.  Not having these qualifying questions should be identified as a limitation within the study.

 * Additionally, analysis on the results shows that respondents agreed moderately with the following conditions: 1) You must be online (60.3%); 2) You must provide your email address (62.3%); 3) You must have a token (56.1%). what is the importance of this - of the 37.7% that are not online, is this because they are restricted by culture, access to internet, have a low levels of awareness?  How will you recommend changes to policy etc from these findings?

*Income, savings and assets differentials among women agripreneurs who accessed digital financial products and those that did not access them*

 * the results of the statistical analysis implies/ demonstrate what the data tells us. **It implies or demonstrates the correct language?**

 * The tests for significant difference between annual savings of participants and non-participants indicated a T-value of 2.479 (p<0.05), which also implies that there was a significant difference in the women agripreneurs’ savings for participants and non-participants in DFPs. 

*Awareness of micro-insurance schemes by women agripreneurs in Southern Nigeria*

 * all donut graphs would be better displayed as a table in the paper

*Participation in micro-insurance schemes by women agripreneurs in Southern Nigeria*

 * results would be better demonstrated in tables

 * Results from Figure 5 shows that majority (95.8%) of female **respondents/agripreneurs** who are accessing...

 * **check wording of this sentence "women heads" also "who are not" does not make sense?**  example wording **Figure 6 shows** that of the **women participants** who are not accessing digital financial products participates none (0%) are participating in micro-insurance schemes, which may indicate a lack of awareness opportunities for women without digital access.

 * Figure 7 shows pooled responses (n=480) from women... **use the total number of responses to assist readers to understand the criteria for the analysis**

 *Only 2.5% of the **(all, some, just agripreneures - identify clearly)** respondents are participating in micro-insurance.

 *A minority of the respondents agreed to the following statements: (1) is a minority; (2) is nearly a quarter of responses and (3) is more than a third of responses **- using "a minority" in this instance is lazy and unrepresentative**

 * Results from Figure 9 shows the perception of women agripreneurs (n= 379) **- when referring to women agripreneurs as a subsample of the data set use the n to assist readers to identify the subset.**

 * Figure 9 data would be better displayed as a table

 * From the findings of this research, the perceptions of these women on micro-insurance could be a major reason why they don’t participate in insurance schemes **- this needs to be a discussion of the findings all together - expand e.g Overall the study identifies XX, YY and ZZ, which clearly identifies that the perceptions of agrientrepreneurial women/women participants poor  participation in insurance schemes.**

**Conclusion**

 *.... must have a bank verification number **and must open** an account **to participate.**

 * This implies that you are more advantaged in using digital finance in business - **is this conclusive? Perhaps those who are more successful are more likely to adopt such products, be cautious coming to singular conclusions.**

 * with the above changes and more thoughtful discussion, the conclusions could be made stronger.

Is the work clearly and accurately presented and does it cite the current literature?

Partly

If applicable, is the statistical analysis and its interpretation appropriate?

Partly

Are all the source data underlying the results available to ensure full reproducibility?

Yes

Is the study design appropriate and is the work technically sound?

Partly

Are the conclusions drawn adequately supported by the results?

Partly

Are sufficient details of methods and analysis provided to allow replication by others?

Yes

Reviewer Expertise:

Technology adoption by women in agriculture, internet access and connectivity, women in agriculture, agricultural marketing research design, delivery and analysis, policy recommendations.

We confirm that we have read this submission and believe that we have an appropriate level of expertise to confirm that it is of an acceptable scientific standard, however we have significant reservations, as outlined above.

# Reviewer report: Reviewer response for version 3

**DOI:** 10.5256/f1000research.159847.r243529

**Published:** 2024-03-06

**Authors:** Wonder Agbenyo

**Affiliations:** 1 Sichuan Agricultural University, Ya'an, Sichuan, China

This topic is interesting and needs a more robust result as compared to the mere basic statistical analysis in the work. 

 I strongly suggest the authors adopt PSM to analyse the impact of digital technology on the financial inclusion of women agripreneurs.  

 What is the moderation between the key variables in this manuscript?

 In general, the background and topic are very educational; however, the statistical approach is not robust enough. 

 *compare assets, income and savings of women agriprenuers with and without digital financial products and services in Southern Nigeria*

 Objective 2 is key to this research hence needs to be well addressed in the study especially with a more robust method.

Is the work clearly and accurately presented and does it cite the current literature?

Yes

If applicable, is the statistical analysis and its interpretation appropriate?

No

Are all the source data underlying the results available to ensure full reproducibility?

No

Is the study design appropriate and is the work technically sound?

Yes

Are the conclusions drawn adequately supported by the results?

Partly

Are sufficient details of methods and analysis provided to allow replication by others?

No

Reviewer Expertise:

Digital finance, Agricultural Economics and econometrics

I confirm that I have read this submission and believe that I have an appropriate level of expertise to confirm that it is of an acceptable scientific standard, however I have significant reservations, as outlined above.

# Reviewer report: Reviewer response for version 2

**DOI:** 10.5256/f1000research.148037.r173819

**Published:** 2023-05-26

**Authors:** Lee-Ying Tay

**Affiliations:** 1 Faculty of Business, Multimedia University, Melaka, Malaysia

I have reviewed the article and am pleased to share that this article is approved for indexing after careful consideration.

Is the work clearly and accurately presented and does it cite the current literature?

Partly

If applicable, is the statistical analysis and its interpretation appropriate?

Yes

Are all the source data underlying the results available to ensure full reproducibility?

No source data required

Is the study design appropriate and is the work technically sound?

Partly

Are the conclusions drawn adequately supported by the results?

Partly

Are sufficient details of methods and analysis provided to allow replication by others?

Partly

Reviewer Expertise:

Area of research: Digital finance, Stock price, Digital financial products and services

I confirm that I have read this submission and believe that I have an appropriate level of expertise to confirm that it is of an acceptable scientific standard.

# Response

**Published:** 2023-12-06

**Authors:** ugwuja vivian

**Affiliations:** University of Port Harcourt, Nigeria

Thank you so much.

# Reviewer report: Reviewer response for version 1

**DOI:** 10.5256/f1000research.134069.r170322

**Published:** 2023-05-02

**Authors:** Jean-Claude KOULADOUM

**Affiliations:** 1 Department of Economics, University of Moundou, Moundou, Chad

**Thank you for giving me this opportunity to review this paper**.

**This paper is entitled as**: Leveraging on digital technology for financial inclusion of women agripreneurs in Southern Nigeria

**The objective conducts** by the authors are very interesting and the method used to achieve this is appropriate. Reading this article raises some questions:

1. Why is the case of Southern Nigeria important to study in the relationship between digital technologies and financial inclusion? Your general introduction does not provide this information.
2. Your general introduction contains almost no authors. This is surprising.
3. The literature review appears incomplete, is not critical, nor is there sufficient academic support for the arguments in the article. You can provide at the beginning of this section the theoretical and empirical foundation of digital technology for financial inclusion.
4. The policy recommendation part needs to be revised. 
5. References to previous research: Either compare your results with the findings from other studies or use the studies to support a claim. This can include re-visiting key sources already cited in your literature review section or saving them from citing later in the discussion section if they are more important to compare with your results instead of being a part of the general literature review of prior research used to provide context and background information.

Consideration of the above comments will improve the quality of this article.

Is the work clearly and accurately presented and does it cite the current literature?

No

If applicable, is the statistical analysis and its interpretation appropriate?

Partly

Are all the source data underlying the results available to ensure full reproducibility?

Partly

Is the study design appropriate and is the work technically sound?

Partly

Are the conclusions drawn adequately supported by the results?

Partly

Are sufficient details of methods and analysis provided to allow replication by others?

Partly

Reviewer Expertise:

NA

I confirm that I have read this submission and believe that I have an appropriate level of expertise to confirm that it is of an acceptable scientific standard, however I have significant reservations, as outlined above.

# Response

**Published:** 2023-05-07

**Authors:** ugwuja vivian

**Affiliations:** University of Port Harcourt, Nigeria

1. Why is the case of Southern Nigeria important to study in the relationship between digital technologies and financial inclusion? Your general introduction does not provide this information.

Reply: I will attend to that

1. Your general introduction contains almost no authors. This is surprising.

I am surprised to see this comment, because I have more than 5 authors in the introduction

1. The literature review appears incomplete, is not critical, nor is there sufficient academic support for the arguments in the article. You can provide at the beginning of this section the theoretical and empirical foundation of digital technology for financial inclusion.

Reply: I will do that

1. The policy recommendation part needs to be revised. \

Reply: I will attend to that

1. References to previous research: Either compare your results with the findings from other studies or use the studies to support a claim. This can include re-visiting key sources already cited in your literature review section or saving them from citing later in the discussion section if they are more important to compare with your results instead of being a part of the general literature review of prior research used to provide context and background information.

Reply: The previous study was cited twice, in the literature review and discussion section

# Reviewer report: Reviewer response for version 1

**DOI:** 10.5256/f1000research.134069.r151576

**Published:** 2022-10-04

**Authors:** Lee-Ying Tay, Gek Siang Tan

**Affiliations:** 1 Faculty of Business, Multimedia University, Melaka, Malaysia

Overall, the manuscript is satisfactory with improvement needed. The comments are as below:

**Is the work clearly and accurately presented and does it cite the current literature?**

 Answer: Partly. The manuscript should add a section of literature review discussing the latest issues on (1) digital technology for financial inclusion; (2) evidence of financial inclusion of women agripreneurs; (3) variables of interest including income, savings, and others. The authors should also cite sufficient and updated references in recent five years.

**Is the study design appropriate and is the work technically sound?**

 Answer: Partly. The authors should add in research questions that are consistently mapped with research objectives. The authors should also add a short write-up on how the research questions and objectives are mapped since the research objective (1) and (3) are achieved through descriptive analysis.

**Are sufficient details of methods and analysis provided to allow replication by others?**

 Answer: The total sample size is not consistent in ‘’Abstract” and ‘’Methodology”. Please further describe the formation of the sample size. It would be great if the authors can attach the survey instruments in this manuscript.

**Are the conclusions drawn adequately supported by the results?**

 Answer: Reasoning should be given why men agripreneurs were not included in the study and provide suggestions for future research on how men agripreneurs can be studied.

Is the work clearly and accurately presented and does it cite the current literature?

Partly

If applicable, is the statistical analysis and its interpretation appropriate?

Yes

Are all the source data underlying the results available to ensure full reproducibility?

No source data required

Is the study design appropriate and is the work technically sound?

Partly

Are the conclusions drawn adequately supported by the results?

Partly

Are sufficient details of methods and analysis provided to allow replication by others?

Partly

Reviewer Expertise:

Area of research:  Digital finance, Stock price, Digital financial products and services

We confirm that we have read this submission and believe that we have an appropriate level of expertise to confirm that it is of an acceptable scientific standard, however we have significant reservations, as outlined above.

# Response

**Published:** 2023-12-06

**Authors:** ugwuja vivian

**Affiliations:** University of Port Harcourt, Nigeria

Sorry for responding late, I  will make all the necessary corrections as pointed out by the reviewer

# Response

**Published:** 2023-12-12

**Authors:** ugwuja vivian

**Affiliations:** University of Port Harcourt, Nigeria

I have made all the necessary corrections.
